# In search of lost fleas: reconsidering Paul-Louis Simond’s contribution to the study of the propagation of plague

**DOI:** 10.1017/mdh.2022.19

**Published:** 2022-07

**Authors:** Christos Lynteris

**Affiliations:** Department of Social Anthropology, University of St Andrews, 71 North Street, St Andrews KY16 9HL, Scotland, UK

**Keywords:** Plague, Institut Pasteur, Rat, Flea, Experiment, India

## Abstract

Paul-Louis Simond’s 1898 experiment demonstrating fleas as the vector of plague is today recognised as one of the breakthrough moments in modern epidemiology, as it established the insect-borne transmission of plague. Providing the first exhaustive examination of primary sources from the Institut Pasteur’s 1897–98 ‘India Mission’, including Simond’s notebooks, experiment carnets and correspondence, and cross-examining this material with colonial medical sources from the first years of the third plague pandemic in British India, the article demonstrates that Simond’s engagement with the question of the propagation of plague was much more complex and ambiguous than the teleological story reproduced in established historical works suggests. On the one hand, the article reveals that the famous 1898 experiment was botched, and that Simond’s misreported its ambiguous findings for the *Annales de l’Institut Pasteur.* On the other hand, the article shows that, in the course of his ‘India Mission’, Simond framed rats as involved in the propagation of plague irreducibly in their relation to other potential sources of infection and not simply in terms of a parasitological mechanism. The article illuminates Simond’s complex epidemiological reasoning about plague transmission, situating it within its proper colonial and epistemological context, and argues for a new historical gaze on the rat as an ‘epidemiological dividual’, which highlights the relational and contingent nature of epidemiological framings of the animal during the third plague pandemic.

A rat captured in a plague-infested district of colonial Karachi is placed inside a large glass jar. To add to the already existing number of fleas hosted by the animal, twenty more, captured on a cat, are thrown into the jar. Twenty-four hours later, the rat is already close to dying. At that point, a small iron cage containing a second rat is lowered into the jar. One side of the cage is covered in wire mesh. Soon after, the first rat dies, but is removed only thirty-six hours later. The autopsy certifies it died of plague. Left inside the cage in the jar, the second rat also dies on the fifth day of the experiment. Having been originally healthy, its autopsy certifies plague.^1^ The conclusion is that the second rat could have only been infected by means of the fleas carried by the first rat, ‘not only because a wire mesh separated the animal in the experiment from the plague rat, but above all because we have never succeeded in infecting a rat or a mouse by placing them in contact with animals inoculated in the laboratory and free from parasites’.^2^

Few experiments are better known in epidemiology than the one published in the *Annales de l’Institut Pasteur* in October 1898 by the Pasteurian doctor Paul-Louis Simond, proving that rat fleas are vectors of the plague-causing bacterium known today as *Yersinia pestis.* Coupled with clinical observations of a blister (*phlyctène*) found in human plague patients, which Simond attributed to flea bites, it is an experiment that is today widely recognised as marking a pivotal step towards the ‘conquest’ of plague.^3^ Indeed the Institut Pasteur annually celebrates the day, June 2, on its Twitter account.^4^ The combination of utter simplicity and its ability to finally identify the transmission pathway of one of the most dreaded diseases in human history have made Simond’s experiment one of these iconic moments when a simple experimental design and a great idea come together to produce a true scientific breakthrough.

Or that is what we have been led to believe by accounts based on Simond’s original description of the experiment (1898) and its longer recollection nearly forty years later (1936).^5^ If the 1898 article was not immediately recognised as bringing about the breakthrough that Simond and the Institut Pasteur had been undoubtedly hoping for, the experiment became gradually solidified in medicine’s experimental and hero-scientist pantheon following the Second World War.^6^ This Eureka moment has been enshrined not only in institutional histories of the Institut Pasteur, but also in scholarly historical works, which have unreservedly lauded Simond’s discovery: ‘Doctor Paul-Louis Simond casts a giant shadow on the destiny of mankind. He lifted the veil that until then had covered the mysterious means of plague transmission’, wrote Edward Crawford in the definitive article on Simond’s plague research.^7^

And yet, surprisingly, to date no historical account of this iconic discovery has gone back to the archive to examine Simond’s experimental notes and ask: What led Simond to undertake his famous experiment? What ideas did Simond hold about rats, fleas and plague transmission at the time? What was the place of Simond’s rat and flea studies within his broader research on plague in British India, and within the context of colonial medicine and the third plague pandemic? And what evidence do we have of his famous experiment, as well as other experiments with rats or their fleas from his notes and correspondence?

While in recent years histories of scientific networks, technicians and laboratory subalterns have provided an indispensible turn in the examination of colonial medicine, the decolonisation of medical historiography has also relied on the development of critical approaches to established and institutional histories of the life and work of ‘great discoverers’ in the field, such as Ronald Ross, Patrick Manson, Wu Liande, Carlos Chagas and others. But whereas the life and work of key figures of the Institut Pasteur’s colonial project, like Alexandre Yersin and Charles Nicolle, have come under sustained scrutiny, this has not been the case for Simond.

Through a cross-examination of Simond’s 1898 article, fieldnotes and correspondence, in this article, I provide a critical re-examination of Simond’s approach to rats and their role in the propagation of plague in the course of the Institut Pasteur’s ‘India Mission’ (1897–98). The findings of the article challenge established histories of Simond’s research on two levels. First, they demonstrate that Simond’s experimental notes cast serious doubt on the validity of the famous rat/flea experiment, and reveal Simond’s knowledge of and frustration with this fact. Second, by moving away from a singular focus on the June 1898 rat/flea experiment, they show that Simond maintained a much more complex engagement with question of the relation between rats and plague, which accommodated different transmission pathways and modes of propagation of the disease, beyond those mediated by fleas. This re-examination of Simond’s research permits us to see how evidence uncritically accepted as conclusive for more than a century were in fact less evident at the time of their production, and that this evidentiary uncertainty or contingency needs to be taken seriously if we are to understand events as clouded by decades of scientific doxa as Simond’s supposed discovery of the transmission pathway of plague.^8^ Second, my re-examination of Simond’s plague research shows that, during the initial outbreaks of the third plague pandemic in British India, the rat emerged as a focus of epidemiological reasoning not as an infectious disease host in and of itself, but as what, borrowing from anthropological theory, I will call an ‘epidemiological dividual’. In other words, that the rat was instituted as a propagator of plague and as a target of colonial public health intervention through complex ways of relating it to other potential sources of infection. Through this examination of Simond’s plague research in British India, we thus arrive at a new understanding of the most celebrated engagement on the part of Pasteurian medicine with the question of the propagation of plague, and at a new understanding of broader colonial medical framings of the rat as a disease host at the end of the nineteenth century.

## Simond’s India Mission

Born in 1858, Paul-Louis Simond joined the Institut Pasteur in 1895 after studying medicine at Bordeaux and serving as Médecin de 1ère classe des Colonies in French Guyana and in Guangzhouwan (Kouang-Tchéou-Wan), a French treaty port in South China, where he first encountered plague in 1893.^9^ Returning to Paris in 1895, Simond worked in Ilya Ilyich Metchnikoff’s laboratory on the subject of the ‘biology of coccidians parasitising intestinal tissue of rabbits and salamanders’, in the process of which he identified coccidian flagella as associated with the male sex of the organism; something that later contributed to understanding of the ‘life cycle of the malarial parasite’.^10^ He thus combined both colonial medical experience and a pedigree of laboratory work in metropolitan France on parasites alongside one of the most celebrated scientists of his time. Having been appointed to the bacteriological laboratory of Saigon in 1897, Simond was soon called by Émile Roux to replace the discoverer of the plague pathogen, Alexandre Yersin, at the Institut Pasteur’s mission to British India, arriving in the colony in May 1897.^11^

Part of the third plague pandemic (1894–1959), a global pandemic that would lead to ten million deaths in India alone, plague outbreaks in the Indian subcontinent began in September 1896, causing a major public health as well as political crisis.^12^ In the first four years of the epidemic in British India (1896–99), foreign commissions from Italy, Germany, Austria, Egypt, and Russia, as well as by the Institut Pasteur conducted scientific research on the disease.^13^ The Institut Pasteur, France’s leading medical research institution, was at the time heavily invested in the study of plague. The discovery of the plague bacillus by Yersin in Hong Kong (1894) had been a crowning achievement for the Institut Pasteur and for French imperial medicine. A French discovery made on British colonial soil and in competition with Japanese scientists trained by German bacteriologists, the discovery was iconic in the manner in which it set French medicine in a leading position within inter-imperial scientific competition.^14^ Rather than simply leading to a proliferation of Pasteurian research on plague in the context of the third pandemic, however, Yersin’s discovery was quickly followed by the far more ambitious quest for a cure of the disease. The Institut Pasteur’s India Mission, initially led by Yersin, was not aimed at discovering the transmission pathway of plague. Instead it was part of what Aro Velmet has aptly called the ‘Pastorization of plague containment’.^15^ Yersin aimed to test and further develop the anti-plague serum he had invented together with Albert Calmette and Amédée Borrel in Paris in 1895, which Yersin had tested in an outbreak context in Canton and Amoy (China, June–July 1896).^16^ Though designed to cure rather than prevent infection, the serum would soon stand in direct competition with prophylactic methods of plague control within what Pratik Chakrabarti has called the ‘experimental theatre of vaccination’; most importantly the plague vaccine developed for the British in Bombay (1897) by Waldemar Haffkine.^17^ With Yersin returning to Indochina only three months into his India Mission, his replacement, Simond, would spend nearly fourteen months in British India (June 1897 to August 1898, with a break between February 10 and March 2, 1898, when he returned briefly to Saigon), conducting research on the serum in among other places Bombay, Agra, Karad, Mandvi and Karachi.

## The rat as an epidemiological dividual

How did Simond take interest in the subject of rats and their fleas in the context of his India Mission? To answer this question we need to follow rats and their fleas as they appear in Simond’s notebooks and correspondence before and during his experiments with the animals, which commenced in May 1898. Only through this examination of everyday note-taking and note-sharing, and its cross-examination with Simond’s published work, can we begin to understand how he developed his framing of the rat/flea-borne propagation of plague. Though both fieldnotes and letters on the subject are often laconic and rarely if ever continuous, they are our best chance to understand which narratives about the relation between rats, fleas and plague Simond found convincing at the time, and what theories of his own he developed over the course of Institut Pasteur’s India Mission. Whereas the 1898 article presents a teleological narrative, where various angles on the rat’s relation to plague are brought together so as to lead to Simond’s ‘discovery’ of the role of rat fleas in the transmission of the disease, in his fieldnotes and letters we encounter a much more complex, syncopated and ambiguous framing of the rat-plague relation, where fleas appear only fleetingly up until the moment of the experiment that would be the cause of their fame in the context of Simond’s biography and research.

The sources available for reconstituting Simond’s study of and experiments with rats during the India Mission are two notebooks and his correspondence, all held at the archives of the Institut Pasteur in Paris. Of these, Simond’s notebook titled ‘Observ. concern. épid. de peste’ [Observations concerning the plague epidemic] (1897–98, henceforth ‘the carnet’, from the French for notebook, for disambiguation from other notebooks kept by Simond during his India Mission) is the most extensive and richest source and yet also one that has been completely overlooked in historical studies of Simond and plague-related research. The carnet is a squared, cardboard-bound notebook (17.5 × 22 cm) composed of 129 unnumbered pages. It initially takes the form of a dated diary, starting September 1, 1897, but this is interrupted after November 22, 1897. Following that entry, the carnet contains fieldnotes and analyses, longer retrospective discourses and reflections, all intermingled with occasional diary-like notes, some dated and some not. Simond’s day-to-day notes in the carnet stop with his departure for Saigon (February 10, 1898), with Simond providing a summary of his research to that point over fifteen densely written pages, and noting that he spent the twenty days that he had to wait for an Indochina-bound boat by ‘treating some plague patients and completing my observations on the propagation of the disease’.^18^

Simond did not resume his day-to-day note-taking after his return to India in March 1898. Instead, what takes up the rest of the carnet is a long discourse on the propagation of plague. The discourse appears to be a sketch of Simond’s 1898 article, including detailed notes on the rat experiments discussed in the *Annales de l’Institut Pasteur.* ^19^ However, the carnet’s long discourse is not identical to the 1898 article. The two differ on several points, including significant passages of epidemiological argument. The nature of this long discourse is moreover strikingly different to the notes proceeding it in the carnet, as, although clearly being a text in progress, punctuated by extensive deletions, ellipses and corrections, this is a cumulative, synthetic and non-spontaneous text. It is unclear when the text was written; different writing styles suggest that it was produced gradually rather than in one sitting. However, it is likely that this was written as a draft for the 1898 article following a letter by Simond’s patron, Émile Roux, on March 12, 1898 asking him to write a report ‘on the propagation and march of the plague in India – the modes of contagion and prophylaxis for the *Annales*’.^20^ A detailed examination of the carnet would be indispensable for an in-depth historical examination of the Institut Pasteur’s India Mission. However, as the aim of this article is not the latter, but more specifically Simond’s approach to the relation between rats and plague, I will limit my engagement with this source to the examination of the latter.

The carnet opens with a note, which provides the earliest evidence of an interest in rats by Simond during his part in the India Mission and immediately situates it within prevailing colonial medical concerns and ideas about plague in British India at the time: ‘In Karad plague began in [unreadable] we found […] dead rats inside the gr[ain] shops [unreadable] various houses. Karad is a great grain market’.^21^ Further down on the same page, Simond noted (September 16, 1897): ‘In Karad, in ordinary times, there is a great commerce of grains, which explains, it seems to me, due to the great number of rats that exist in this town, that the epidemic develops with such intensity’.^22^ These short notes clearly indicate that, already by September 1897, four months into his expedition, Simond was well immersed in colonial framings of rats in relation to the spread of plague that were prevalent during the first year of the pandemic in British India. Contrary to Simond’s 1898 article, which dismisses British colonial scientific opinions and debates on the role of rats in the transmission of plague as ‘too timid’, as well as historical readings that stress Simond’s opposition to British approaches to the disease, his carnet notes show that he was well aware of the latter’s extent, complexity and importance as regards introducing new understandings of the disease.^23^

Simond’s understanding of the rat’s role in the propagation of plague sat comfortably within prevalent British colonial medical framings of the rat as what we may call an ‘epidemiological dividual’. Borrowing the term from social anthropology, by this I mean that medical works and debates at the time instituted the rat as an epidemiologically knowable and actionable propagator of plague through investigating the complex ways in which it related to other potential hosts or vectors of the disease.^24^ As a heuristic, the ‘dividual’ thus allows us to consider the relational institution and transformation of the rat as a propagator of plague without falling back on the metaphysics of agency implied in Latourian notions of the ‘network’ and the ‘assemblage’.

The potential plague sources in relation to which the rat was instituted and transformed in British colonial medical literature in India during the key epidemic years of 1896 to 1899 included grain, excrement, the soil, cloths, as well as the capacious category of the ‘infected house’. Taking as a common ground the bacteriological nature of plague, as identified in Hong Kong in 1894, not everyone agreed on how these sources or repositories of plague infection interacted or what conditions were necessary for this interaction to produce plague epidemics or to preserve plague over non-epidemic periods. It was, however, always by means of interrogating its relation to such potential hosts or vectors, rather than itself in isolation, that colonial medical experts came to frame the rat; so much so that, as a propagator of plague, the animal became inseparable from the pestigenic relations, real or imaginary, in which it became entangled. What is often portrayed in historiography as a confused period where colonial doctors grappled with explanations of the spread and maintenance of plague in a supposedly inevitable pathway towards clarity, through the discovery of plague’s single and true vector (the rat flea), was in reality an epistemologically intense, nuanced and complex era of plague research where the focus was explicitly and systematically on the pestigenic *relations* between potential so-called ‘agents’ of the disease. To take this dividualist focus of epidemiological reasoning seriously, and not reduce it to a prelude of the discovery of a single vector, is crucial if we are interested in understanding colonial medicine as an epistemological and a biopolitical process that incorporated, integrated and often mutually unsettled bacteriological and sanitary approaches to plague as a disease that remained consistently outside the framework of tropical medicine. Both the epistemic and ‘moral force’, to follow Pratik Chakrabarti’s analysis of bacteriology in British India, this approach was derived not so much from *identifying* the supposed ‘agents’ of plague as from *relating* them and intervening on their pathogenic relations.^25^ This was a process that was politically and epistemologically entangled with but distinct from the process of ‘creating links between germs, climate, culture, and race’ in India.^26^ If the rat was a charismatic player in inter-linking these relational paradigms, it was to a great extent because of what Nicholas Evans has identified as a colonial focus on the animal’s supposedly transgressive character; a focus that allowed the colonial government to integrate bacteriological and sanitarian approaches to plague, blame native customs for the propagation of the disease, and ‘re-establish the fixed racial and caste categories through which it ruled’.^27^

That Simond did not oppose this paradigm but partook in the relational ontology of the rat for the greatest part of his India Mission is evident in his fieldnotes and correspondence, where his key concern was the ‘propagation of plague’; a notion that referred both to the transmission of the disease and to what was generally known at the time as the ‘progress’ of the disease across space. Simond’s 1898 article and the draft contained in his carnet followed various pathways of reasoning about this question, which brought different potential hosts/vectors of plague in relation to one another. In the 1898 article, Simond argued that when imported human cases preceded indigenous ones, a period of more than one month intervened between the two, followed by a ‘period of latency’ during which cases were localised or manifested a seemingly sporadic pattern before developing into a full epidemic.^28^ For Simond, it was during the period linking the latency and the epidemic stage, ‘a period of rapid growth of the epidemic so that it reaches its acute state’, that ‘the possibility of the extension of the plague without a human intermediary appears clearly’.^29^ This argument is not, however, present in the draft of the article contained in the carnet, where another argument is developed in its place. There, Simond portrayed what he called the ‘simultaneous’ infection of people inhabiting a household as a significant epidemiological datum that, he argued, excluded the possibility of human-to-human contagion and could be attributed instead to infection through the soil or items in the household, which had been in turn infected by rats.^30^

For Simond, one of the reasons why rats played a key role in the propagation of plague was their supposed propensity to migrate in response to the disease; a theory that was rapidly gaining ground in British approaches to plague in India at the time, forming an important alternative to theories that attributed the spatial diffusion of plague to human mobility. In an entry to his carnet dated January 16, 1898, Simond noted that the Bombay Plague Commission had ascertained that the death of rats preceded that of humans, and that the former were observed to ‘emigrate’ from Bombay to surrounding villages thus creating new foyers of the disease.^31^ The note directly mentions Thana (Thane, a city just outside Mumbai), which indicates that Simond might have been exposed to the so-called ‘Rat Progress’ theory developed at the time.^32^ One of the phenomena that had perplexed British colonial doctors and sanitary officers studying and trying to control plague in the first year of the pandemic in India (1896–97) was the alleged rapid disappearance of rats soon after the beginning of an outbreak in a given locality. This, some reasoned, resulted from rats migrating under the bane of the epizootic; a movement that could result to the epidemic spreading to new locations.^33^ By the end of 1897, arguments that ‘In towns and villages rats undoubtedly convey the disease from house to house and from district to district’ drew particular support from evidence systematised by the Collector of Thana, A. C. Logan, who argued that the supposed migration of rats led to a successive infection of urban neighbourhoods; a process coined the ‘Rat’s Progress’.^34^

The debate over migrating rats as drivers of the spread of plague over space dominated much of British colonial medical literature on the disease at the time and was of great importance to the colonial management of plague. For—depending on the scale to which it was applied—accepting that rats spread plague over short, middle or long distances rendered corresponding, highly contentious measures of controlling and containing human movement (quarantine, isolation, sanitary cordons) obsolete.^35^ The idea of the ‘migratory rat’ also had an early and lasting impact on Simond’s reasoning regarding the drivers of the propagation of plague. In the carnet’s entry dated January 16, 1898, he described the inspection of a house in the Bombay quarter of Mandvi (not to be confused with the town in Kutch bearing the same name), in July 1897, where two young girls had fallen victim to the disease: ‘We did not see dead rats in the house, but the rats had disappeared 10 or 12 days before’.^36^ Simond concluded that this might be due to the outbreak of plague causing ‘their emigration’.^37^ Commenting on the subject of rat migration in an earlier letter to Émile Roux, he had speculated that rats fled infected locations once noticing ratfalls among them and that, in this way, ‘in short distances (the maximum observed by me is 3 km) plague can be transported by rats without the intermediary of humans’.^38^

This epidemiological reasoning would be clearly formulated in Simond’s later meditations on rats, where he maintained that, in the course of the peak of the epizootic, ‘panic arises, which determines the emigration of the majority of the rats’ thus reducing their numbers in affected areas.^39^ The rat’s alleged migratory nature was consequently used as the key to explain another of what Simond called the ‘mysterious points in the history of the plague’: its disappearance and eventual recrudescence in a given location after a period of time (which Simond calls *acalmie*, or lull).^40^ That in both the carnet’s draft and the 1898 article Simond dismissed the role of the seasons in this process of attenuation and recrudescence is not surprising, as both Pasteurian and British understandings of plague largely excluded it from the frame of tropical diseases. What is, however, significant is that, contrary to what we would expect from a Pasteurian and a collaborator of Yersin, Simond did not resort to the idea that the soil acted as a reservoir of the disease; a theory that his colleague had painstakingly tried to demonstrate in Hong Kong and which formed a distinguishing trait of Pasteurian approaches to plague at the time.^41^ Instead, Simond framed rats as responsible for this phenomenon:

In summary we can presume know that a large part of the rats in a town die during the plague epidemic, that a large part run away and others that a certain proportion of them that remain are immunised having experienced a non-fatal form of plague. If the rat is as we think the principal [written above: essential] propagator of plague, there is reason to admit that a new human epidemic would not be able to break out until young generations of rats susceptible to contract the disease have repopulated the town.^42^

The period of ‘lull’, which Simond saw as a period of a locality’s immunity to plague, was essentially a time when plague could not affect rats.^43^ This was a statement of great practical importance in the context of plague control, for by shifting the focus from the soil to rats, it rendered unnecessary a series of contentious anti-plague methods employed in India at the time, such as deroofing houses or baking the soil of houses whose floor was made of bare ground.^44^

By leaving the soil out of his epidemiological frame, Simond detached himself from Yersin’s thesis, and forged a closer relation between rats and plague than most dividualist frameworks of that relation in operation in British India allowed for at the time. This tacit tendency to epidemiologically individuate the rat, as it were, in its relation to plague becomes more clear when we examine a passage connecting rats to plague’s recrudescence that is present in Simond’s carnet but not in the 1898 article. There Simond speculated that the key reason for the ‘lull’ and subsequent recrudescence of plague in a given locality was not, as Yersin would have it, a move from attenuation to virulence, but what we would today call an ethological transformation:

The natural distrust of the rat may be a reason that the survivors of the epidemic avoid the causes of contamination, it is very likely, for example, that they renounce the habit of eating the corpses of their fellow creatures after having witnessed the mortality resulting from such feasts. It would then be expected that later generations would resume this custom favourable to the extension of the epidemic.^45^

Cannibalism as a source of plague infection among rats had been demonstrated in what formed the first extensive series of experiments with plague and rats in the first half of 1897 by the German Plague Commission to India, whose highly-acclaimed report would be translated by Robert Nathan in the first volume of his authoritative report on plague, commissioned by India’s Home Department, in 1898:

Since it is known that these animals in a state of freedom are accustomed to gnaw the bodies of their companions dead of plague, it is easy to understand that the pestilence must spread very quickly among them and destroy the whole rat-population of a place, and that by means of rats the plague germs can be introduced from one home into another and conveyed to men.^46^

The findings were endorsed by no less than Robert Koch, who in his diary from his own plague expedition to India (1897) commented both on rat cannibalism as a mode of inter-rat transmission and on the infectivity of rats ‘from the uninjured mucous membranes and digestive tracts’.^47^

Simond’s notes on rat cannibalism fall comfortably within this bacteriologically-backed framing of rat-to-rat infection. And yet, we need to take a closer look at what is entailed in this idea of plague’s lull as a result of rats’ ethological transformation. For, being here at the mythic heart of epidemiological reasoning as applied by Pasteurian science to plague, we need to ask with Claude Lévi-Strauss: ‘When a myth attributes to an animal a certain behaviour under determined circumstances, does it follow an empirical observation, or an independent but unfounded belief, or does this attribution result from an internal constraint to the myth?’.^48^

The idea that cannibalism drives rat-to-rat infection and that plague’s recrudescence is the result of rats somehow learning not to cannibalise one another reveals the complex interplay of plague and rat ontologies in Simond’s epidemiological reasoning. On the one hand, plague-driven epizootics were seen as dependent on rat’s supposedly cannibalistic nature; an idea potentially connected to notions of rat cannibalism as a population self-limitation mechanism going back to the early nineteenth century.^49^ On the other hand, rather than plague being simply the result of rat cannibalism, it was actually portrayed as if having a transformative agency upon the latter. For, as a result of observing plague-induced ratfalls, rats were said to suspend their natural ‘character’ and abstain from eating one another, thus preserving themselves from further infection and ending the epizootic. This self-limiting property of plague through working directly on the supposed nature of rats needs to be compared here with the self-propagating faculty of the disease implied in the aforementioned notion of rat migration as a key plague-spreading mechanism. In this case, plague-induced ratfalls were said to cause ‘panic’ among rats, leading them to flee, thus spreading the disease to new locations. Here too plague was portrayed as having a direct ethological impact on rats, which, if perhaps not as profound as that of suspending their supposedly cannibalistic nature, was said to have a significant epidemiological impact. From this perspective, rats were pivotal in plague’s life-cycle insofar as they were transformed by the disease in two distinct ways: the first, behaviour transformation (panic-driven flight) resulted in the propagation of plague into new locations, while the second, ontological transformation (suspending their cannibal nature) led to the quiescence of the disease in the affected location.

Taken together, these notes on rat migration and cannibalism demonstrate that, eight months into his India Mission, Simond, who had endorsed the complex, dividualist framing of rats and plague developed by British colonial doctors, was beginning to form his own framing of rat’s relation to plague. This innovatively proposed plague’s supposedly transformative impact on rats as a *sine qua non* of both the propagation and the self-limitation of the disease. However, this framing of rats’ supposedly special relation to plague did not yet challenge the overall dividual character of the relation, which was to a great extent reliant on the idea of transmission through the alimentary tract. Simond’s cannibalism theory clearly implicated the latter in the propagation of the disease. Yet, contrary to what his publications allow us to surmise, this was not the only manner in which he incorporated the alimentary mode of transmission into his epidemiological reasoning.

## Plague by the alimentary tract

By 1898 the idea that the alimentary tract was a conduit of plague had received sustained attention by medical and sanitary experts, forming a fertile ground of what following David Barnes we may call a sanitary-bacteriological synthesis on plague causation.^50^ There is no space here to develop the history of this theory in detail, but it is important to note that it was endorsed by pivots of plague-related expertise such as Alexandre Yersin, Robert Koch, and James Cantlie, while also leading to comments and debates in the daily press in India.^51^ The theory of alimentary infection received particular support in a report published in 1897 by the Staff-Surgeon Wilm of the Imperial German Navy on plague in Hong Kong in the preceding year.^52^ Also available in three instalments and as a single, digest article in the *Indian Medical Gazette*, Wilm’s thesis was widely read, referenced and discussed.^53^ The observation that plague was ‘most frequently’ contracted ‘by way of the alimentary tract’ was, in Wilm’s opinion, ‘substantiated by the results of experiment on animals’, as well as by post-mortems of human victims showing significant impact on the stomach and mesenteric glands.^54^

In his 1898 article, Simond attacked the theory of alimentary infection unequivocally, presenting his discovery of the flea vector as the only viable alternative. By contrast, his fieldnotes indicate a far more receptive approach to the idea. Indeed, as we have already seen (the case of Karad), a key entry-point for Simond’s interest in rats was their connection to grain – an object that was intricately connected to questions regarding the relation between rats and plague in the course of the first years of the pandemic in the Indian subcontinent. A key concern for colonial authorities was that infected rats might introduce plague into grain depots where they were attracted for nourishment. There, it was feared, they could spread plague to healthy rats, as well as contaminate grain and gunny bags, rendering these into ‘sources of conveyance of the disease to human beings’.^55^

Grain had been experimentally tested for plague in the first half of 1897 by the leading British bacteriologist in India, Ernest Hanbury Hankin. In a letter dated February 19, 1897, Hankin stated that he ‘never succeeded in detecting’ plague in grain.^56^ Nonetheless, framings of grain as implicated in the spread of the disease remained prevalent and were further fuelled by the question of how plague was introduced to Karachi during the city’s second epidemic of the disease (1898). Reports and testimonies given to the Indian Plague Commission (1898–99) maintained that dead rats (which had been conspicuously absent in the first outbreak between December 1896 and July 1897) were found in gunny bags and in grain houses and mills.^57^ The majority of these bags had reportedly arrived from Bombay, fuelling the question of the inter-relation between grain and rats in introducing and spreading the disease to new locations, as well as a debate on what measures became necessary if this mode of plague propagation was indeed in place.^58^

Simond agreed with the plausibility of this theory, allowing also for the infection of Karachi through the direct importation of rats from Bombay.^59^ In a short discussion of the plague outbreak in Haridwar in the spring–summer of 1897, he also speculated on the reasons why at the end of the epidemic in the great pilgrimage city, an epizootic of rats was observed in nearby Kankhal (both in today’s Uttarakhand).^60^ He reasoned that the disease spread through infected foodstuff being transported from ‘infected houses’ in Haridwar to Kankhal, where they ‘were eaten by rats and spread the ep.[idemic] among them’.^61^ In a letter to Roux (October 22, 1897), Simond clarified that it was not grain per se that he considered as a possible host of plague, but rat excrement soiling it:

In Bombay, grain was accused of being the source of the epidemic and people of importance claimed that it was neither rats nor microbes but wheat and rice that transmitted plague. There is an element of truth in this ignorant reasoning, deriving from the droppings of sick rats, the microbe must frequently use grain as a vehicle, and in humid weather as a growing medium.^62^

Furthermore, in a manner that amplified the dividual entanglement of the rat as propagator of plague, Simond speculated that there may also be an excrement-mediated airborne pathway between rats, grain and humans: ‘The person who grinds the grain, by inhaling the cloud of flour that forms around the grindstone, has a good chance, if the grain has been soiled by plague-infected rats, to absorb the microbe through the lungs’.^63^

It is thus clear that, contrary to the tone of a priori dismissal carried in his 1898 article, Simond was acutely interested in the question of so-called external sources of plague, with his epidemiological reasoning being very much attuned to British colonial medical debates on the subject; a question that furthermore offered itself as a lucrative field for experimentation. In order to test the ‘principal hypothesis that the microbe exists in the exterior milieu’, Simond proceeded with conducting a series of experiments, largely reproducing designs previously employed by British and German bacteriologists.^64^ Of these, the twelve contained in his carnet and in the 1898 article are identical.^65^ Of the twelve, nine focused on testing different modes of alimentary infection. They involved feeding rats and mice with agar preparations containing plague bacilli (Experiments I, II), organs (Experiments III, V, VIII), blood (Experiments III, IV), and cadavers of plague-infected rats and mice (Experiment III), as well as human bloody expectorations (Experiment VI), and grain mixed with human urine of a plague patient (Experiment VII). Additionally, Simond performed two alimentary-related experiments with other animals, repeating Experiment VII with a monkey (Experiment IX), and feeding a ground squirrel (*rat palmiste*) with bread soaked in the blood of a rat dead from plague (Experiment XII). All animals, with the exception of Experiment IV, survived without contracting plague.

Experiment IV involved the inoculation of a rat with plague bacteria. The rat was then placed inside a glass jar containing grain. After its death, the rat was removed and another rat was placed in the jar among the grain, which was now ‘soiled by the excretions of rat no. 1’; the rat was kept there for five days without manifesting plague.^66^ Yet, when a third rat was given to drink the blood of rat no.1 diluted in water, it died of plague within four hours. However, Simond dismissed this evidence, arguing that the fact that rat no. 3 had a wound on its lower lip and that it developed a cervical bubo indicated an infection through the wound rather than through the alimentary tract.

It was the findings of these experiments that, according to Simond’s article and carnet, led him to seek other possible transmission pathways, and in particular one that involved ‘parasitism’.

## Fleas in the archive

In his carnet Simond noted that, following the experiments showing the ineffectiveness of the alimentary tract in plague infection, there needed to be ‘a natural means of penetration of the microbe into the epidermis apart from the accidental lymphothetic excoriations admitted until now’, and that this was no other than parasitism: ‘the flea is the parasite which by its habits seemed to us a priori capable of playing the role of inoculator’.^67^ But just how a priori was this assumption in his epidemiological reasoning?

Interest in insects as possible insect vectors of plague involved an expansive list of species ever since Yersin, in the article announcing the discovery of plague’s bacterium, mentioned his suspicion of flies as spreaders of the disease.^68^ As Rohan Deb Roy has argued, insects and ‘parasites’ ‘constituted a vibrant social category’ in colonial contexts that was often used to racialise and generate class, gender and caste hierarchies around infection and its supposed sources.^69^ As shown most prominently through the historiography of the identification of the mosquito as malaria’s vector, insects also formed a lucrative field for medical research and for scientific competition that was more often than not underlined by inter-imperial antagonism.^70^ While experiments on insect-borne plague infection preceded Ronald Ross’s discovery of the mosquito as a vector of malaria (August 20, 1897), it is reasonable to assume that the latter further catalysed interest in establishing an insect vector for plague. However, by contrast to malaria-focused research, investigations around plague’s potential insect vectors were not undertaken within the framework of tropical medicine, or, at this stage, through an ecological lens focused on the relations between insects and the environment.

When it comes to Simond’s interest in rat fleas, the archive is much less replete than in relation to rats or the alimentary tract. The first note on the possibility of fleas being involved in plague transmission appears in an undated entry, made before January 16, 1898, on the ninth page of the carnet. The note simply reads: ‘And the possibility of contamination by rat fleas’.^71^ The single-line note is located in a half-page section of Simond’s carnet, titled ‘Plague’, which is composed of eight similarly short, one- and two-line-long, notes. These laconic notes are for the most part abbreviated, making it difficult to be always sure about their intended meaning, but in their vast majority they concern rat-to-rat plague transmission, through rat faeces and urine, cannibalism and grain.

The purpose of these notes is not clear, yet it is evident that, by mid-January 1898, Simond was considering fleas as a mode of rat-to-rat plague transmission among others. On the other hand, no discussion of this potential transmission pathway is present in his correspondence at the time. The only other indication of an interest in the subject, prior to the June 1898 experiment is a short bibliographical note on page 37 of his carnet.^72^ This stand-alone note, positioned at the top of a page left otherwise blank, is significant as it mentions two key works on plague transmission by means of insects, in the following order: George H. F. Nuttall’s ‘On the role of animals in the spread of plague’, and Ogata Masanori’s ‘On the plague epidemic in Formosa’, both published in 1897 in the reputable German medical journal *Centralblatt für Bakteriologie und Parasitenkunde.* ^73^

The article by Nuttall, an American-British bacteriologist and pioneer of insect-borne parasitology, contained a detailed comparative review of research on the susceptibility of different animals to plague. He further noted a series of experiments he conducted in order to establish the involvement of flies and bedbugs in plague transmission. Nuttall stated that experiments showed that flies died of plague and that they could ‘live for several days, after they have received the infected food, and one cannot therefore deny that they can play a role in the spread of plague, when they fall into foodstuffs or leave their excrement on them’.^74^ By contrast, he found that ‘plague bacilli eventually die out in bedbugs’ rendering them improbable transmitters of the disease.^75^ Though crucial at the time for its systematic review of insect-borne transmission, Nuttall’s article made little mention of fleas.

Ogata conducted his research in response to an outbreak of plague in Taiwan, which was at the time a Japanese colony. The epidemic had broken out in October 1896 and by June 1897 had led to forty-five deaths. Ogata, who was in charge of bacteriological investigations, reported that during his stay in Taihoku he ‘had 6 dead rats at my disposal. Two were brought to me by another doctor, two I found on the street, one came from the barracks and one was brought by a sanitation officer’.^76^ What follows is a fascinating account of interspecies encounter in a laboratory setting:

Since on opening the package I saw some fleas on the animals, one rat was immediately wrapped up again, the other was doused with a 1/1000 sublimate solution, whereby 20 fleas were caught. This rat had in the middle of its tail a crust the size of a rice grain, by removing which one saw a deep ulcer covered by pus.^77^

Pure cultures of plague being delivered, Ogata proceeded with catching fifteen fleas from the second rat, which was brought in sterile water: ‘Seven of the fleas caught in the sterile water were now crushed between two sterilised glasses and two mice were inoculated with them. One of these mice died after three days’.^78^ Ogata’s conclusion was that fleas could carry plague and spread it to animals, as well as potentially humans. ‘It is possible’, Ogata concluded, ‘that plague is actually (as the story of the illness in Formosa [Taiwan] taught us) first a rat pest, and this animal is the nearest cause of the spread of this disease to humans’.^79^

Though Simond’s note on the two works is very short, it crucially mentions that Ogata had shown that ‘fleas from dead rats transmit plague’.^80^ Historians’ negation of or doubts regarding Simond’s knowledge of Ogata’s work at the time of his experiments with rats and fleas are thus completely unjustified and unfounded.^81^ While Simond did not mention him in his 1898 article, he was clearly aware of Ogata’s research and of the fact that it was him who had first shown the transmission of plague by means of fleas. Moreover, the placement of the note in the carnet shows that Simond knew this at the time of undertaking his own rat and flea experiments.

The only other source indicating Simond’s engagement with the rat-flea hypothesis before his summer 1898 experiments comes from his correspondence with Hankin. In his letters to Simond (the letters by Simond to Hankin do not survive in the archive) the British bacteriologist brings up rats as early as May 18, 1898, when he mentions his experiments in Haridwar showing an attenuation of the plague bacterium by means of subsequent passages through the animals.^82^ As extensively discussed in his testimony to the Indian Plague Commission, Hankin speculated that this could mean that rats could move plague from place to place but not conserve it in any specific location.^83^ The suspected inability of rats to act as reservoirs of plague in inter-epidemic intervals caused Hankin to seek an alternative host. Hankin put the question to Simond thus:

But what does the microbe do during this long incubation period in the locality? Not likely that it passes through the rats, because by that process it becomes attenuated. Perhaps in some other animal it finds the conditions to restore its virulence. Maybe an insect, the fly? Please tell me what you think about these ideas. I hope to be able to experiment on this point’.^84^

Like Nuttall, Hankin was interested in ascertaining the infectivity of bedbugs, and to test this he used rats, which he injected with crushed bedbugs caught on or in the vicinity of hospitalised plague patients in Bombay.^85^ Of three rats thus inoculated in the course of experiments conducted in the first half of 1897 only ‘one was found to have died of plague’.^86^ Hankin was also interested in the infectivity of ants, reflecting wider colonial concerns with the insects in India at the time.^87^ In a ‘Note on the relation of insects and rats to the spread of plague’, published in the same issue of *Centralblatt für Bakteriologie und Parasitenkunde* as Nuttall’s article, he concluded that after ‘a long series of researches’ he found that ants ‘neither die of the disease [n]or retain the infection for any time’.^88^ However, Hankin reasoned that by eating rats that had died of plague, and ‘by thus distributing and carrying about infected material they may increase the risk of infection from dead rats’, thus implicating the alimentary tract in insect-borne interspecies plague transmission.^89^

In his correspondence with Simond over May 1898, Hankin dwelt at length on rat experiments aimed at ascertaining the attenuation of plague in rats. The design followed by Hankin in the experiment mentioned in his letter to Simond dated May 31, 1898 is significant, for it greatly resembled that used by Simond in the aforementioned Experiment IV and for his famous, June 2, rat/flea experiment. It involved placing four/five healthy rats in a cage alongside the cadaver of a rat that had been artificially infected with plague:

Gradually after many delays (some weeks) these rats died, without any sign of plague. I replaced the rats with others and I finally obtained one dead rat with very few microbes having the visual character of plague in its organs. I did not succeed in cultivating this. It is possible that if I had continued to place rats in this cage I would have had an infection with virulent plague. But this is an isolated experiment. It is possible that the fleas have played a part. Can you not repeat it?^90^

Given that the letter is dated May 31 and was sent to Simond (who was at the time in Karachi) from Agra, it is possible but by no means certain that it reached him before June 2, when Simond’s famous experiment is said to have taken place. And yet, as da Silva has pointed out, the letter shows that the question of flea transmission was common among plague researchers at the time, who also shared experimental notes and designs in an effort to elucidate the question.^91^

If flea-borne plague transmission played a role in Simond’s epidemiological reasoning in the course of the India Mission, up until the summer 1898 experiments this certainly did not form an ‘a priori’. Rather, fleas were simply part of the expansive array of hosts and vectors that, in their contingent and relational entanglements, instituted the conditions necessary for the propagation of plague. What appears to have led Simond to experiment with fleas is the combination of three elements: his failure to demonstrate alimentary infection, having recently read Ogata’s article, and possibly having received Hankin’s encouragement to that direction. Simond’s 1898 article suggests that his clinical observations of a blister (*phlyctène*) in human victims made him suspect insect-borne infection, with fleas being among the possible candidates. As there are no notes in Simond’s archives showing the in-progress collection of data on this clinical framework, it is not possible to ascertain if the blister theory was developed before the flea experiments, acting as an incentive, or if it followed them, as a clinical support to what Simond knew were rather doubtful experimental results.

## Simond’s flea experiments

I will now examine the famous experiment of June 2, 1898.^92^ Whereas Simond’s carnet draft and his 1898 article contain identical descriptions of all other experiments, the description of the experiment purportedly demonstrating plague’s transmission between rats by means of fleas bears striking differences between the two sources. We read, also transcribing crossed-out words in the manuscript as these contain valuable information:

The most important experiment is that on the transmission of plague by means of living fleas from a plague-affected rat. I only had the opportunity to execute this experiment once. A sick rat that was captured in the town of Mandvi where the plague reigned is and looked sick died in the cage where I had placed it. The examination of the rat cadaver [unreadable] after at the time of death demonstrated to me allowed me to observe the presence of a few fleas on it. The cadaver was placed at the bottom of a large jar, covered with a sheet of paper placed and above that on the sheet I placed a small cage made of a wooden box of which a single side was lined with a iron-wire mesh and containing three young rats aged one and a half months. After 24h the canvas which covered the jar was lifted, I noticed at this moment that several fleas that were on the internal wall of the vase had escaped and the rat’s cadaver was removed. The small cage containing the three young rats remained another 48 hours in the jar, after which it was removed. One of the young rats died of plague 5 days later. This experiment seems convincing to us. However, as it was impossible for us to find the fleas that probably escaped from the jar during the manipulations, it is desirable that the opportunity missed here be repeated.^93^

Whereas the published experiment described Simond placing a living rat, suspected of being naturally infected with plague, in the experimental jar, in the experiment contained in his carnet it is clear that the rat died in a cage before being placed in the jar.^94^ No cat providing additional fleas to those found in the original rat is mentioned in the carnet. The carnet by contrast mentions that the rat’s cadaver was first covered in a sheet of paper, something not mentioned in the article. More importantly, what was then placed in the jar, on top of the paper-covered dead rat, was not ‘a small iron cage’ but a ‘wooden box’ whose one side was ‘lined with a iron-wire mesh’. There is no indication that this was suspended inside the jar; instead it is most likely (the crossed-out notes make this ambiguous) that it was placed directly on top of the paper sheet covering the dead rat. Moreover, the box contained not one, but three rats. Then the experiment faced quite a predicable problem: to remove the dead rat’s cadaver (twenty-four and not thirty-six hours later, as the article claims), Simond lifted the canvas covering the experimental apparatus and all fleas escaped. The experiment was in other words botched. Nonetheless, Simond did not interrupt the experiment, but left the three rats inside the box contained in the jar for another forty-eight hours. Of the three rats, only one died, on the fifth day. The fleas having escaped, it was impossible to test them for plague.

It is extremely unlikely that these notes do not correspond to the experiment described in the 1898 article, as they are positioned in Simond’s notebook in-between other notes on the question of the fleas and clinical observations of the flea-inflicted blister, which are copied verbatim or with slight stylistic revisions in the article. Moreover, nor the experiment as described in the article nor any other similar experiment or attempt to replicate it are recorded in the carnet or in a second notebook, titled ‘P H Experiments’, where Simond noted down further seventy-four experiments conducted between May 25 and July 25, 1898.^95^

There are two possibilities. First, that the experiment in Simond’s 1898 article did take place as described in the article, but was not noted down, or that the notes were taken down in a notebook other than the two existing ones, which was subsequently destroyed or lost. Second, and most probable, that Simond purposefully misreported what was in fact a botched and inconclusive experiment into an ‘ideal’ one, which unambiguously proved the transmission of plague between rats by means of fleas. We should here pay attention to the short paragraph following the description of the experiment in Simond’s 1898 article:

The experiment repeated under the same conditions gave us success in a mouse that died of plague in three days, and two failures in adult rats. The dexterity with which these, while we observed them, defended themselves against the attack of the fleas and destroyed them by eating them, make us think that this is a frequent reason for the failure of the experiment.^96^

In the 1898 article, Simond furthermore mentions that in another experiment a rat that had died of plague had its fleas removed and was then placed in a jar with seven healthy rats, none of which was infected.^97^ These additional experiments were not recorded either in the carnet, the ‘P H Experiments’ notebook, or any other surviving archival source. In his letter to Roux dated August 31, 1898 Simond by contrast claimed to have had success in an experiment with one rat and in another with a mouse. These contradicting claims may indicate a way for Simond to be creative with facts: by extracting from his published description of the June 2 experiment the two rats that did not contract plague and by then claiming that these were part of some other attempt to replicate the supposedly successful experiment, Simond might have hoped to conceal the fact that the actual experiment was inconclusive and at the same time balance the failure to produce plague in these two cases by inventing yet two more replications of the experiment, this time successful ones. In this way, the Eureka experiment could be rhetorically salvaged and future failures to replicate it could be pre-empted as coincidental or due to rats’ supposed habit of eating the fleas infesting them, something that if true would have certainly been noted in either of his laboratory notebooks.

In a crossed-out paragraph in Simond’s carnet we read: ‘We do not claim that the experiments we have had already made provide indisputable proof of this mode of transmission. But this is strikingly corroborating, as is the clinical observation’.^98^ In another passage in the carnet, also not copied in the article, Simond further expressed his hesitation, by mounting an apagogical defence: ‘While none of the present clinical or experimental observations are material evidence of parasite transmission, all of them appear to leave little room for any other modes’.^99^ Simond’s uncertainty about his findings is most evident in his letter to Roux on August 31, 1898 where the Archimedean feeling of euphoria expressed in Simond’s publications is singularly absent. Simond wrote that he was not ‘able to carry out absolutely irrefutable experiments so far’, adding: ‘I am very anxious to hear your feelings about this. Of course you will do with this study of the propagation what you like, I wrote it to submit it to you as a last resort, whatever your judgement and you may publish it or not, I will not feel any stupid sting of self-esteem’.^100^

That Simond did not believe the experiments transcribed in his article were conclusive is evident from the fact that, after June 2, he conducted a further sixty-six experiments on plague transmission. These, together with eight more experiments conducted between May 25 and June 2, are contained in the ‘P H Experiments’ notebook.^101^ All seventy-four experiments are carefully noted and dated, yet none is discussed in his 1898 article. The majority of these experiments involved the inoculation of mice, rats, pigeons and monkeys with plague cultures or the triturate of organs of infected animals. The notebook also contains twelve experiments with fleas, all conducted after June 2, of which seven involved the injection of crushed fleas into healthy rats or mice, and five experiments with live fleas.

On the one hand, Simond conducted five stand-alone experiments with fleas, mice and rats between June 30 and July 7, 1898. The first experiment (June 30) was an immediate failure, echoing the trouble with the famous June 2 experiment: Simond placed a young rat in a jar with five fleas extracted from another that had died of plague, but before the experiment could proceed the fleas managed to escape.^102^ The same day, rather than trying to repeat the experiment, Simond injected a white mouse with a flea taken from a dead rat, diluted in water. Although the rat died on the night between July 9 and 10, an examination of its corpse revealed no plague bacilli.^103^ Simond would conduct two more stand-alone experiments with fleas, on July 1 and 7, involving the injection of the debris of a flea extracted from a plague-infected rat into a rat and a mouse respectively, without however recording the results.^104^ Finally, on July 7 he injected a white mouse with a trituration of two fleas taken from a plague-infected rat. The mouse died soon after, on the night between July 7 and 8, but the post-mortem proved inconclusive with the animal’s spleen showing many bacilli, ‘where some resembled B.P [the plague bacillus]’ – a statement to be contrasted with the usual note ‘stuffed with B.P’ or ‘B.P in abundance’ marking cases where Simond actually identified the plague bacillus in experiments contained in the notebook.^105^ These experiments thus failed to show a connection between fleas and plague transmission between rats, even by direct injection, which may be a further reason why Simond was privately not at all certain that his June 2 experiment had conclusively proven the efficacy of flea-borne transmission.

At the same time, Simond conducted composite experiments involving a range of transmission pathways. Given that the notebook never attempts to present a systematic picture of these experimental chains (experiments are noted down in chronological order), it is more likely that experiments progressed in an unplanned, on-the-go, case-by-base manner. For reasons of space, it is not possible to present these experimental chains here in any detail. It is, however, indicative that one chain involved seven mice and eight rats, which Simond proceeded to cross-infect between July 14 and August 1, 1898, employing multiple infection pathways: plague culture inoculation, inoculation with triturations of infected organs and triturations of fleas, as well as confinement with rodent cadavers and with fleas. While the experiments led to no conclusive results, this veritable web of infection indicates that, throughout the summer of 1898, Simond, on the one hand, retained his interest in non-flea-related plague transmission, while, on the other hand, privately maintaining a focus on the rat as an epidemiological dividual: an animal whose role in the propagation of plague could only be understood in terms of its relations with other potential hosts and transmission pathways of the disease.

## An accomplished Pasteurian

Understanding Simond’s preoccupation with the question of the propagation of plague, his interest in rats and their fleas, as well as the decision to publicly oppose dividualist framings of plague, seek a singular source of the disease, and claim the discovery of plague’s insect vector in spite of his failure to produce a successful experiment need to take into account, first, how Simond engaged with and was informed by framings of disease causation at the time, and, second, how colonial structures and strategies of power fostered this public move from *relating* to *identifying* plague’s ‘agents’.

As shown in this article, Simond’s engagement with bacteriology, entomology and parasitology was unsystematic and in many cases opportunistic. His research notes, correspondence and publications evince good knowledge of current ideas and studies about plague transmission, but more often than not no systematic engagement with them. On the one hand, following Lukas Engelmann’s approach to plague research in the first decades of the third pandemic, we may say that Simond often engaged in ‘epidemiological casuistry’: fitting observed facts with suitable theories so as to support his argument at different stages of its development irrespective of whether the theories used were compatible or not.^106^ On the other hand, we need to take Simond ethnographically seriously as an innovator in terms of disease causation, to the extent that he self-staged himself as such and engaged in a public performance of laboratory (though not always systematically bacteriological) experimentation.^107^

Second, in terms of an analytical framework focused on colonial power, the implementation of rat-control measures as part of anti-plague policy in British India preceded Simond’s research by two years. On the one hand, Simond’s supposed discovery had no immediate impact on the dividualist epistemological framework informing these policies, on colonial approaches to rats in the context of plague control, or on plague control policy in India more broadly.^108^ It would take more than a decade for these to shift so as to focus singularly on rats and their fleas, as the combined result of new technologies focused on exterminating these animals (fumigation, poisoning, ratproofing) and of research conducted by the joint plague commission of the Royal Society and the Lister Institute.^109^ To assume, as histories of the third pandemic commonly do, that Simond’s discovery was the watershed leading to these shifts is fallacious. On the other hand, returning to the point raised above about taking Simond’s self-presentation as a scientific innovator seriously, we need to consider the stage of colonial antagonism where this Pasteurian self was performed.

As mentioned in the first part of this article, Simond had been dispatched to India not in order to study how plague spread, but in order to test the anti-plague serum developed by the Institut Pasteur and prove its efficacy. In other words, his mission was part of what Chakrabarti has aptly called the Institut Pasteur’s ‘moral crusade’ against diseases in the colonies; a process predicated upon the production of a sense of ‘hope and optimism’ that ‘the abstract, multiple, and complex causalities of disease in the tropics could have a singular cure or prevention’.^110^ We need to follow da Silva’s analysis of Simond’s serum research in order to understand that Simond’s rat/flea research unfolded in a context that was causing embarrassment to and pressure on the Institut Pasteur.^111^ This was because, by early 1898, it was becoming increasingly clear that the serum faced a range of problems. Da Silva’s meticulous examination of Simond’s research has shown that, by the end of his mission, he was unable to prove the efficacy of the serum.^112^ In a report on his tests with plague patients in Karachi, Simond admitted that these had left much to be desired: 55% of the patients treated with the serum succumbed to the disease, marking a staggering difference from Yersin’s previous tests in Canton (1896) where only 6% of patients given the serum had died of plague.^113^

This presented a significant economic problem for the Institut Pasteur, as the serum was at the time one of its key global prospective commodities, which was moreover in direct competition with other plague prophylactics, most importantly Haffkine’s vaccine.^114^ But it was also a problem of imperial prestige. For, having previously claimed the discovery of plague’ pathogen, over the competing claim by Japan’s Kitasato Shibasaburō, the Institut Pasteur was now also aiming to claim the discovery of the cure and/or prevention of the disease.^115^ In short, as ‘a diplomatic strategy centred on the production and distribution of the anti-plague serum’, by 1898, the Institut Pasteur’s imperial ambition stood severely challenged by the actual limitations of its product.^116^ If the aim of the India Mission was not just to establish and improve the efficacy of the serum, but also to showcase it to the world, then, for all Simond’s optimism that progress was in sight, this was a major scientific, economic and PR debacle for the Institut Pasteur.

If by March 1898 Simond had clear indications that he would not be able to showcase the serum’s efficacy, it is reasonable to assume that he might have seen an opportunity to salvage the Institut Pasteur’s reputation and his own career by presenting a discovery that continued the one made by Yersin four years earlier, and mirrored the identification of mosquitoes as the vectors of malaria by Ronald Ross. However, Simond was well aware that he was not alone in this race for plague’s insect vector. As we have already seen, he had read Ogata’s 1897 article, which was the first to actually demonstrate a rat-to-rat transmission of plague by means of fleas. He was also aware that Hankin was keenly interested in this transmission pathway, and in co-authoring an article on the subject with him, an invitation he shunned. Finding himself in the make-or-break point of his career, it is possible that these factors led Simond to take the perilous leap into misrepresenting his experimental results. All the more so as success in this field was not simply a matter of choice but something urgently demanded by his Institut Pasteur patrons.

We have already seen that Simond’s 1898 article was not something spontaneously written as a result of the June experiments, but a publication requested by Émile Roux, in March that year, with the expressed directive of discussing the propagation of the disease in India.^117^ No archives survive on the specifics of Roux’s decision to ask Simond to compose this report in his March 12 letter. Such published reports were often part of Pasteurian missions. It is also clear from the preceding and subsequent letters by Roux to Simond that the co-founder of the Institut Pasteur was gradually accepting the failure of the serum and was seeking a way to redeem the India Mission and the prestige of Pasteurian science more broadly. As da Silva has convincingly argued, Simond would have felt under immense pressure to produce a breakthrough that would salvage both the reputation of the Institute and his own career.^118^ Did Simond choose to risk publishing what he knew was a botched experiment so as to save face and please his superiors? Did he interpret Roux’s tacit signals for an opportunity to follow Ross’s identification of the mosquito as a vector of malaria and the identification of ticks as vectors of Texas Fever by claiming the discovery of plague’s insect vector for the Institut Pasteur?^119^ Or was he becoming worried of being overtaken from within the Institut following news from Nha Trang that Yersin—in an apparent effort to cover up a leak from his lab—was framing white ants as propagators of plague in the course of a local outbreak in July–August 1898?^120^ Whereas Simond’s claim of discovery did not receive the immediate international recognition he was probably hoping for, if his primary aim was to appease his superiors, the overall review of his India Mission and of the 1898 article by Roux suggests at least an internal success. Not only the failure in establishing the effectiveness of the serum was not attributed to him, but, receiving particular praise for his contribution to the understanding of the propagation of plague, Simond was lauded as ‘a good bacteriologist, a good epidemiologist and a good diplomat – in one word an accomplished Pasteurian’.^121^ Simond would be rewarded by being appointed director of the Institut Pasteur in Saigon (1898–1901), received several awards and honours in relation to his discovery, and would soon be invited to lead other research missions in relation to insect vectors.^122^ If the Institut Pasteur’s envoy could not fulfil the promise of Pasteurianism as ‘as a mode of knowledge that combined causation and cure within a singular paradigm to provide a new moral force in resolving the myriad realities of what were known as colonial pathologies’, he could at least by a sleight of hand deliver part of this ideological kernel by publicly marking a shift away from the dividualist paradigm of British colonial medicine as regards the propagation of plague, and announcing in a dramatic manner the true and only vector of the disease.^123^

## Conclusion

In this article I have examined how, in the course of his part in the Institut Pasteur’s India Mission (1897–98), Paul-Louis Simond approached the rat’s involvement in the propagation of plague. Rather than this being limited to the rat’s flea, or a parasitological mechanism (as presented in his publications and accepted in historical works so far) my examination of Simond’s archives has shown that the Pasteurian doctor developed complex theories and frameworks about the propagation of plague, largely premised upon the rat’s ontology as an epidemiological dividual. In the first eight months of his mission, Simond focused on rats’ relations to other potential propagators of plague, as well as on the role of rat cannibalism in the geographic spread and self-limitation of plague. Upon his return from Saigon in March 1898, prompted by a request by Roux to publish a report on the propagation of plague, he conducted a series of experiments on plague transmission. By August 1, he had conducted at least eighty-six experiments on different transmission pathways, some involving extremely complex multi-pathway chains of infection. Of these, he eventually chose to publicly put his trust in a single experiment involving live-flea transmission between rats. Simond publicly dismissed all other transmission pathways, although several of his experiments showed these to be at least as viable as flea-borne infection.

Simond’s famous experiment with fleas was botched and inconclusive, as its design was faulty and as two out of three rats did not contract plague. The archives also reveal that Simond was aware of these severe limitations of the experiment and that he continued to conduct experiments after the supposed discovery of June 2, 1898. These formed the largest number of experiments on the transmission of plague during his mission (sixty-six out of eighty-six), and show that Simond remained privately interested in other transmission pathways. However, Simond opted not to publish these experiments in his 1898 article. He moreover did not repeat the experiment of June 2, which he eventually misreported in the *Annales de l’Institut Pasteur* so as to claim the discovery of the flea-borne pathway of plague, while knowing that this had already been demonstrated a year earlier by the Japanese bacteriologist Ogata.

The reason why Simond chose to misreport his experimental results with rat-fleas, and not publish on the greatest number of experiments he conducted on the transmission of plague cannot be securely ascertained, as direct information about this decision is not available in the archives. It is, however, probable, as shown in this article, that Simond felt under immense pressure to produce some sort of breakthrough as a result of the failure of the actual objective of his India Mission, which was to prove and showcase the efficacy of the Institut Pasteur’s plague serum. Seen in the context the wider international scientific competition for identifying insect vectors of infectious diseases, the specific international scientific competition for identifying plague’s insect vector, and the Institut Pasteur’s already established prestige in plague research, Simond’s decision to abruptly shift from a relational framing of plague’s ‘agents’ to one focused on the identification of a single insect vector and his move to publicly misrepresent what he knew was a botched experiment are not surprising. Forming part of a career as a doctor within an internally and externally competitive environment for great discoveries, Simond’s approach to rats and their fleas did not simply rhyme with the broader ambitions and agendas of what Aro Velmet has called ‘Pasteur’s Empire’, it in fact formed a key element to it, as the glorification of his June 2 discovery has come to show ever since.^124^

More than simply marking a troubling contribution to the historiography of Pasteurian science, the findings of this article indicate the need to develop a new direction in the historical examination of epidemiological framings of the rat. For rather than being an individual animal species which could be singularly blamed for plague in the context of the emergency of the third plague pandemic, I have shown that, at least in the initial years of the pandemic, the rat was instituted as a propagator of the disease only in its relations with other suspected hosts or vectors. If for epidemiologists at the turn of the century the rat was inseparable from these relations and unintelligible outside them, the historical examination of plague-related science and epidemic control needs to move away from its hitherto held rat/flea framework, and focus instead on epistemologies and biopolitics of the inter-institution of potential sources and transmission pathways of plague in the context of the third pandemic.

## Footnotes

1

Paul-Louis Simond , ‘La propagation de la peste’, Annales de l’Institut Pasteur, 12 (1898), 625–687 , 674. All translations in this article are mine.

2
*Ibid.*, 675.

3

Edward A. Crawford Jr., ‘Paul-Louis Simond and His Work on Plague’, Perspectives in Biology and Medicine, 39 (1996), 446–458 , 457; Frédérique Audoin-Rouzeau, *Les chemins de la peste: Le rat, la puce et l’homme* (Rennes: Presses Universitaires de Rennes, 2003); Henri H. Mollaret , ‘La découverte par Paul-Louis Simond du rôle de la puce dans la transmission de la peste’, Revue practicien, 41 (1991), 1947–521925381; Marc Simond , Margaret L. Godley and Pierre D. E. Mouriquand , ‘Paul-Louis Simond and His Discovery of Plague Transmission by Rat Fleas: A Centenary’, Journal of the Royal Society of Medicine, 91 (1998), 101–104 960275510.1177/014107689809100219PMC1296502; M. Simonet , ‘Paul-Louis Simond, un épidémiologiste et microbiologiste méconnu’, Feuillets de biologie, 335 (2017), 43–48 .

4 For example, https://twitter.com/institutpasteur/status/870597545847926784.

5 Paul-Louis Simond, ‘Comment fut mis en évidence le rôle de la puce dans la transmission de la peste’, *Revue d’hygiène*, 58 (1936), 5–17.

6 The reception of Simond’s article is not the topic of this article; for a discussion see: Crawford, *op. cit.* (note 3). On the construction of Pasteurian hero-scientists see: Pnina G. Abir-Am, ‘How Scientists View their Heroes: Some Remarks on the Mechanism of Myth Construction’, *Journal of the History of Biology*, 15, 2 (Summer 1982), 281–315; Anne Marie Moulin, ‘Patriarchal Science: The Network of the Overseas Pasteur Institutes’, in Patrick Petitjean, Catherine Jami, and Anne Marie Moulin (eds.), *Science and Empires. Historical Studies about Scientific Development and European Expansion* (Springer Science, 1992), 307–322; Aro Velmet, *Pasteur’s Empire. Bacteriology and Politics in France, Its Colonies, and the World* (Oxford: Oxford University Press, 2020).

7 Crawford, *op. cit.* (note 3), 457. The key institutional history of the discovery is: Mollaret, *op. cit.* (note 3).

8 On making evidence less evident as a goal of anthropological approaches to medicine and public health see: Didier Fassin, *Les mondes de la santé publique. Excursions anthropologiques. Cours au Collège de France 2020–2021* (Paris: Seuil, 2021).

9 Simond et al., *op. cit.* (note 3); Crawford, *op. cit.* (note 3), 447.

10
*Ibid.*, 447

11 Simond et al., *op. cit.* (note 3). The Institut Pasteur laboratory at Nha Trang, Annam, was largely dedicated to the production of the Institut’s anti-plague serum; Velmet, *op. cit.* (note 6).

12

Myron Echenberg , Plague Ports . The Global Urban Impact of Bubonic Plague, 1894–1901 (New York: New York University Press, 2007).

13
*Ibid.*

14 Velmet, *op .cit.* (note 6); Robert Peckham , ‘Matshed Laboratory: Colonies, Cultures, and Bacteriology’, in Robert Peckham and David M. Pomfret (eds.), Imperial Contagions: Medicine, Hygiene, and Cultures of Planning in Asia (Hong Kong: Hong Kong University Press, 2013), 123–147 .

15 Velmet, *op. cit.* (note 6), 31.

16 Pratik Chakrabarti, *Bacteriology in British India: Laboratory Medicine and the Tropics* (Rochester: University of Rochester Press, 2012); Matheus Alves Duarte da Silva, ‘Quand la peste connectait le monde: production et circulation de savoirs microbiologiques entre Brésil, Inde et France (1894–1922)’. Thesis: École des Hautes Études en Sciences Sociales, 2020.

17 Chakrabarti, *op. cit.* (note 16), 49.

18 Archives scientifiques CeRIS – Institut Pasteur (henceforth AIP), SIM.2, A3/81–84, ‘Observ. concern. épid. de peste’.

19 Simond included notes in his carnet draft on where to add such and such paragraph, presumably referring to his 1898 article.

20 AIP, SIM.4, A3/81–84, 37 lett. et cart. d’Emile Roux à Paul-Louis Simond, 105 p. ms., 46 f., Roux to Simond, March 12, 1898.

21 AIP, SIM.2, A3/81–84, *op. cit.* (note 18). Neither in his notes nor in his 1898 article did Simond specify what species of rats he was talking about or experimenting with.

22
*Ibid.*

23 Simond, *op. cit.* (note 1), 640. Velmet, *op. cit.* (note 6) overemphasises Simond’s opposition to British approaches.

24

Marilyn Strathern , The Gender of the Gift: Problems with Women and Problems with Society in Melanesia (Berkeley: University of California Press, 1988).

25 Chakrabarti, *op. cit.* (note 16), 23.

26
*Ibid.*, 6.

27

Nicholas H. A. Evans , ‘Blaming the Rat? Accounting for Plague in Colonial Indian Medicine’, Medicine Anthropology Theory, 5, 3 (2018), 15–42, 37.

28 Simond, *op. cit.* (note 1), 637.

29
*Ibid.*, 637.

30 AIP, SIM.2, A3/81–84, *op. cit.* (note 18).

31
*Ibid.*

32 The term is not mentioned by Simond. Couchman (see note 34) may have been a possible source for Simond, as it included a copy and discussion of Logan’s research.

33 See for example, Surgeon Lieutenant-Colonel T. S. Weir’s ‘Abstract of Report of the Health Officer of Bombay City for 1896’, in: House of Commons Parliamentary Papers (henceforth HCPP), C.9111 Report of Sanitary Measures in India in 1896–1897, Vol. XXX. Simond discussed Weir’s research on other topics in his carnet.

34 See correspondence between A. C. Logan and F. S. P. Lely (Thána, July 12, 1897) in: Malcolm Edward Couchman, *Account of Plague Administration in the Bombay Presidency from September 1896 till May 1897* (Bombay: Government Central Press, 1897), 113.

35 Samuel Cohn Jr, ‘Plague in India: Contagion, Quarantine, and the Transmission of Scientific Knowledge,’ in Christos Lynteris (ed.), *Plague Image and Imagination from Medieval to Modern Times* (London: Palgrave Macmillan, 2021), 191–203.

36 AIP, SIM.2, A3/81–84, *op. cit.* (note 18).

37
*Ibid.*

38 AIP, IND.A1, 4/151–153, 7 lett. de Paul-Louis Simond à Emile Roux, Simond to Roux, October 22, 1897.

39 Simond, *op. cit.* (note 1), 651.

40
*Ibid.*, 657. Also identical in AIP, SIM.2, A3/81–84, *op. cit.* (note 18).

41 Christos Lynteris, ‘A “Suitable Soil”: Plague’s Urban Breeding Grounds at the Dawn of the Third Pandemic’, *Medical History*, 61, 3 (July 2017), 343–357.

42 AIP, SIM.2, A3/81–84, *op. cit.* (note 18). The narrative is reproduced slightly revised in Simond, *op. cit.* (note 1), 656.

43 Ideas of rats developing immunity to plague were hotly debated at the time; see, for example, the testimony of Lieutenant H. J. Walton (IMS) to the Indian Plague Commission; HCPP, Cd.140, Indian Plague Commission, 1898–99. Minutes of evidence taken by the Indian Plague Commission with appendices. Vol. II. Evidence taken from 11th January 1899 to 8th February 1899.

44 Lynteris, *op. cit.* (note 41).

45 AIP, SIM.2, A3/81–84, *op. cit.* (note 18).

46 Robert Nathan, *The Plague 1896, 1897,* Vol. 1 (Simla: Government Central Printing Office, 1898), 27. For the original see: Georg Theodor August Gaffky, Richard Pfeiffer, Adolf Dieudonné, and Georg Sticker, *Bericht uber die Thatigkeit der zur Erforschung der Pest im Jahre 1897 nach Indien entsandten Kommission* (Berlin: Julius Springer, 1899), 57.

47 Robert Koch, ‘Reiseberichte über Rinderpest, Bubonenpest in Indien und Afrika, Ttetse- oder Surrakrankheit, Texasfieber, tropische Malaria, Schwarzwasserfieber’, in G. Gaffky and E. Pfuhl (eds.), *Gesammelte Werke von Robert Koch* (Leipzig: Georg Thieme, 1912), 688–742, 710. Note: this was originally published under the homonymous title in 1898, Berlin, by Julius Springer. The diary entry is ‘Bombay, May 26, 1897’.

48 Claude Lévi-Strauss in: Raymond Bellour, ‘Entretien avec Claude Lévi-Strauss’, in Raymond Bellour and Catherine Clément (eds.), *Claude Lévi-Strauss* (Paris: Gallimard, 1979), 157–210, 172.

49

Neil Pemberton , ‘The Rat-Catcher’s Prank: Interspecies Cunningness and Scavenging in Henry Mayhew’s London’, Journal of Victorian Culture, 19, 4 (2014): 520–535 .

50

David S. Barnes , The Great Stink of Paris and the Nineteenth-Century Struggle against Filth and Germs (Baltimore: Johns Hopkins University Press, 2006).

51 See, for example, Anon., ‘How to Avoid the Plague,’ *The Times of India* (January 12, 1897), 6.

52 M. Wilm, *A Report on the Epidemic of Bubonic Plague at Hong Kong in the Year 1896*, translated by Maurice Eden Paul (Hong Kong: Noronha & Co., 1897).

53 It appeared serialised under the title ‘A Report on the Epidemic of Bubonic Plague at Hong Kong in the Year 1896’ in the *Indian Medical Gazette* issues of May, June and July 1897, and in summary in: M. Wilm, ‘Report on Plague,’ *Indian Medical Gazette,* 32, 4 (April 1897), 137–140.

54 Wilm, *op. cit* (note 52), 27.

55 J. S. Wilkins, ‘Report of the plague epidemic in Cutch-Mandvi’, appearing in: W. F. Gatacre (ed.), *Report on the Bubonic Plague in Bombay 1896–1897* (Bombay: Times of India Steam Press, 1897), 231.

56 S. Comfort and E. H. Hankin, ‘India. The Bubonic Plague Bacillus in Grain and Flour’, *Public Health Reports*, 12, 21 (May 21, 1897), 507.

57 HCPP, Cd.139, Indian Plague Commission, 1898–99. Minutes of evidence taken by the Indian Plague Commission with appendices. Vol. I. Evidence taken from 29th November 1898 to 5th January 1899, 143 (see, Cadell, Dec. 14, 1898).

58
*Ibid.*

59 AIP, SIM.2, A3/81–84, *op. cit.* (note 18).

60
*Ibid.*, undated, between November 22, 1897 and January 16, 1898.

61
*Ibid.* It is possible that Simond drew this information from Hankin’s 1897 article, where he described his experiences with the Kankhal outbreak and his examination of rats between June 15 and 28, 1897.

62 AIP, IND.A1, 4/151–153, *op. cit.* (note 38), Simond to Roux, October 22, 1897.

63
*Ibid.*

64 Simond, *op. cit.* (note 1), 658.

65
*Ibid.*, 658. Three further experiments were conducted on animals with the Institut’s serum.

66
*Ibid.*, 661.

67 AIP, SIM.2, A3/81–84, *op. cit.* (note 18).

68

Alexandre Yersin , ‘La peste bubonique à Hong Kong’, Annales de l’Institut Pasteur, 8 (1894), 662–667 .

69

Rohan Deb Roy , Malarial Subjects: Empire, Medicine and Nonhumans in British India, 1820–1909 (Cambridge: Cambridge University Press), 253 29400918.

70

Jeanne Guillemin , ‘Choosing Scientific Patrimony: Sir Ronald Ross, Alphonse Laveran, and the Mosquito‐Vector Hypothesis for Malaria’, Journal of the History of Medicine and Allied Sciences, 57, 4 (October 2002), 385–409 1518201710.1093/jhmas/57.4.385.

71 AIP, SIM.2, A3/81–84, *op. cit.* (note 18).

72
*Ibid.* The note is undated and there are no surrounding dated notes that may allow its safe dating. The last previously noted date on the carnet is April 1898. Immediately following the note, on the opposite page of the carnet, begins the draft of the 1898 article.

73

George H. F. Nuttall , ‘Zur Aufklärung der Rolle, welche die Insekten bei der Verbreitung der Pest spielen – Über die Empfindlichkeit verschiedener Tiere für dieselbe’, Centralblatt für Bakteriologie und Parasitenkunde, 22 (1897), 87–97 ; Masanori Ogata , ‘Über die Pestepidemie in Formosa’, Centralblatt für Bakteriologie und Parasitenkunde, 21 (1897), 769–777 .

74 Nuttall, ‘Zur Aufklärung der Rolle, welche die Insekten bei der Verbreitung der Pest spielen’, 90, in the original the passage is typed in emphasis.

75
*Ibid.*, 92.

76 Ogata, ‘Über die Pestepidemie in Formosa’, 774.

77
*Ibid.*, 774.

78
*Ibid.*, 775.

79
*Ibid.*, 776.

80 AIP, SIM.2, A3/81–84, *op. cit.* (note 18).

81 Crawford, *op. cit.* (note 3); Audoin-Rouzeau, *op. cit.* (note 3).

82 AIP, SIM.5, A3/81–84, Mission de la peste aux Indes (1897–1898), 12 lett. d’Hankin à Paul-Louis Simond, 1898–1899, Hankin to Simond, May 18, 1898.

83 HCPP, Cd.140, *op. cit.* (note 43). I would like to thank Matheus Alves Duarte da Silva for generously sharing authorised transcripts of this correspondence.

84 AIP, SIM.5, A3/81–84, *op. cit.* (note 82), Hankin to Simond, May 18, 1898.

85 British Library (BL), IOR-V-27-856-1, E. H. Hankin-Investigations on Plague.

86
*Ibid.*

87 Hankin told the Indian Plague Commission that his interest in ants had been elicited by Robert Koch; HCPP, Cd.140, *op. cit.* (note 43), 3. Ants formed part of broader colonial anxieties in India; Rohan Deb Roy, ‘White Ants, Empire and Entomo-Politics in South Asia’, *The Historical Journal*, 63, 2 (March 2020), 411–436. White ants were also framed by Yersin as spreading plague in Nha Trang in an attempt to exonerate his lab from accusations of being the source of a plague outbreak in the summer of 1898; Christos Lynteris ‘Vagabond Microbes, Leaky Laboratories and Epidemic Mapping: Alexandre Yersin and the 1898 Plague Epidemic in Nha Trang’, Social History of Medicine, 34, 1 (February 2021), 190–213 .

88

Ernest Hanbury. Hankin , ‘Note on the Relation of Insects and Rats to the Spread of Plague’, Centralblatt für Bakteriologie und Parasitenkunde, 22 (1897), 437–438 , 437. For a description of the experiments see: BL, IOR-V-27-856-1, *op. cit.* (note 85).

89 Hankin, *op. cit.* (note 88), 437. These experiments were not mentioned in Hankin’s letters to Simond, though their publication in the same issue as Nuttall’s 1897 article may safely lead us to assume that Simond had read them.

90 AIP, SIM.5, A3/81–84, *op. cit.* (note 82), Hankin to Simond, May 31, 1898.

91 da Silva, *op. cit.* (note 16). Simond’s article did not acknowledge his correspondence with Hankin. Simond ignored Hankin’s interest in co-authoring an article on the subject, and published the article without notifying him. Hankin only found about the article being in print by Roux; AIP, SIM.5, A3/81–84, *op. cit.* (note 82), Hankin to Simond, October 25, 1898.

92 The dating of this experiment is noted in neither the 1898 article nor the carnet. Instead it relies on Simond’s 1936 recollection of his experiment. This is supported only by a short note in a small, untitled diary Simond kept during his India Mission, mainly containing travel dates and purchase notes: ‘Experiments on the transmission of plague. Success 2 June 1998’ (Simond often misnumbered 1898 as 1998 in his notes). The note is not made in the proper chronological order for June 2, but at the end of the page; AIP, SIM.2, A3/81–84, carnet de Paul-Louis Simond: ‘1 malle buffle’.

93 AIP, SIM.2, A3/81–84, *op. cit.* (note 18).

94 The use of a jar in this and preceding experiments could have been inspired by Yersin’s earlier effort to demonstrate that plague was ‘contagious’ by placing an infected and a healthy mouse inside a bottle. The experiment is described in Yersin’s article announcing the discovery of the plague bacillus, but not the fact that a bottle was used, something however mentioned in his third report to the General Governor of Indochina (July 17, 1894); Yersin, *op. cit.* (note 68); AIP, IND.A1, 4/151–153, rapport d’Alexandre Yersin au Gouverneur général de l’Indochine, 2 f. ms., Hong Kong, 17/07/1894.

95 AIP, SIM.6, A3/81–84, carnet de Paul-Louis Simond: ‘P H Expériences’. The experiment listed under June 2 in this notebook did not involve fleas.

96 Simond, *op. cit.* (note 1), 674.

97
*Ibid.*, 675.

98 AIP, SIM.2, A3/81–84, *op. cit.* (note 18).

99
*Ibid.*

100 AIP, IND.A1, 4/151–153, *op. cit.* (note 38), Simond to Roux, August 31, 1898. I would like to thank Matheus Alves Duarte da Silva for sharing a copy of this letter. That Roux would not oppose the publication of such inconclusive results should not surprise us, given his own history in falsifying evidence in the famous Rouyer rabies case (1886–87); I would like to thank Ilana Löwy for this information; Ilana Ilana Löwy , ‘Cultures de bactériologie en France, 1880–1900: la paillasse et la politique’, Gesnerus, 67, 2 (2010), 188–216 21425657.

101 AIP, SIM.6, A3/81–84, *op. cit.* (note 95); it is not clear what ‘P H’ refers to.

102
*Ibid.*

103
*Ibid.*

104
*Ibid.*

105
*Ibid.*

106

Lukas Engelmann , ‘Making a Model Plague: Paper Technologies and Epidemiological Casuistry in the Early Twentieth Century’, in Christos Lynteris (ed.), Plague Image and Imagination from Medieval to Modern Times (London: Palgrave Macmillan, 2021), 235–266 .

107

Lorraine Daston and Peter Galison , Objectivity (Cambridge: Zone Books, 2007). For a broader discussion of Pasteurian self-presentation in relation to plague-related discovery, see, Velmet, *op. cit* (note 6); Peckham *op. cit.* (note 14).

108 For a discussion of the reception of Simond’s experiment by the Indian Plague Commission, of measures against rats and fleas proposed by Simond, and a comparison to British colonial measures, see, da Silva, *op. cit.* (note 16).

109

Lukas Engelmann and Christos Lynteris , Sulphuric Utopias: A History of Maritime Fumigation (Cambridge: MIT Press, 2020); Evans, *op. cit.* (note 27).

110 Chakrabarti, *op. cit.* (note 16), 13.

111 da Silva, *op. cit.* (note 16).

112
*Ibid.*

113
*Ibid.* For the published version of the report, see Paul-Louis Simond , ‘Rapport sur les cas de peste traités dans l’Inde Anglaise par le moyen du sérum antipesteux préparé à l’Institut Pasteur de Paris par M. le Dr Roux,’ Annales d’hygiène et de médecine coloniales, 1 (1898), 349–361 .

114 On the Institut Pasteur’s broader economic and industrial history as an imperial history see: Velmet, *op. cit.* (note 6).

115

David J. Bibel and T.E. Chen , ‘Diagnosis of Plague: An Analysis of the Yersin-Kitasato Controversy’, Bacteriological Reviews, 40, 3 (September 1976), 633–651 1087910.1128/br.40.3.633-651.1976PMC413974.

116 da Silva, *op. cit.* (note 16), 119.

117 AIP, SIM.4, A3/81–84, 37, *op. cit.* (note 20), Roux to Simond, March 12, 1898.

118 da Silva, *op. cit* (note 16) and personal communication. Just over a week after the initial demand, Roux would remind Simond to send him ‘that note that I asked you for on the march and the way of being [*manière d’être*] of plague in India’; AIP, SIM.4, A3/81–84, 37 *op. cit.* (note 20), Roux to Simond, March 20, 1898.

119
*Ibid.*, Roux to Simond, August 21, 1898, postscript. The identification of ticks as vectors of Texas Fever was attributed by Roux to Koch, when in fact it was made by Theobald Smith. Neither Roux nor Simond seem to have been aware of or interested in David Bruce’s trypanosomiasis insect transmission research.

120
*Ibid.*, Roux to Simond, 21 August 1898. For details on the Yersin’s ant theory see Lynteris, *op. cit.* (note 88).

121 AIP, SIM.4, A3/81–84, 37, *op. cit.* (note 20), Roux to Simond, September 23, 1898. On Roux praising the *Annales* article as a ‘grand succès’ see *Ibid.*, Roux to Simond, [undated] December 1898.

122 For a discussion of Simond’s later experiments on the transgenerational transmission of yellow fever between mosquitos in Brazil, and the transformation of inconclusive evidence into epidemiological proofs see: Ilana Löwy , ‘The Best Possible Intentions. Testing Prophylactic Approaches on Humans in Developing Countries’, American Journal of Public Health, 10, 2 (February 2013), 226–237 10.2105/AJPH.2012.300901PMC355876923237168.

123 Chakrabarti, *op. cit.* (note 16), 23.

124 Velmet, *op. cit.* (note 6).

